# Hair cortisol concentrations as marker of chronic stress in wild roe deer (*Capreolus capreolus*)

**DOI:** 10.1007/s11259-026-11106-6

**Published:** 2026-02-07

**Authors:** S. Comazzi, V. Vilardo, S. Luridiana, R. Rossi, M. Nobile, A. Giordano, S. Panseri

**Affiliations:** 1 Department of Veterinary Medicine and Animal Science, University of Milan, Lodi, Italy; 2 Department of Veterinary Medicine, University of Sassari, Sassari, Italy; 3 Piacenza Wildlife Rescue Center, Loc Trebbiola, Niviano, PC Italy

**Keywords:** ELISA, Chronic stress, Roe deer, Hair, Cortisol

## Abstract

The stress response to different stimuli involves the release of mediators, with cortisol being the principal, aimed to induce physiological, metabolic, and behavioral changes for restoring allostatic balance. However, prolonged or excessive cortisol secretion may impair welfare by altering immunity, growth, and reproduction. Cortisol concentrations or its metabolites can be measured in several biological matrices, and may accumulate in the hair shaft over periods ranging from weeks to months, making hair a valid matrix for assessing chronic stress. Nevertheless, a validation of this method in roe deer is currently lacking. The objective of this study is to validate an ELISA test for the measurement of cortisol concentration in roe deer hair. Additionally, the effects of age, sex, environmental conditions, and seasonal variations on hair cortisol concentrations are evaluated. Materials and Methods. Hair samples were collected from 45 European roe deer housed in a wildlife rescue center. Cortisol was extracted from hair using methanol, and concentrations were quantified using a commercially available competitive ELISA kit originally developed for salivary cortisol. Results. Hair cortisol concentrations in roe deer ranged from 0.7 pg/mg to 27.25 pg/mg, exhibiting a distinctive bimodal distribution. Factors influencing hair cortisol levels included the age class and the season of collection, with particular emphasis on the rutting season in adults. No significant differences were observed with regard to the type of environment or the cause of rescue. Conclusion. The ELISA method provides a rapid, cost-effective, and reliable approach for assessing long- term alterations in glucocorticoid concentrations in roe deer, yielding results comparable to other more sensitive techniques.

**Supplementary Information:**

The online version contains supplementary material available at 10.1007/s11259-026-11106-6.

## Introduction

Stress is a complex physiological phenomenon triggered by various stimuli and generally classified as acute or chronic. Acute stress is characterized by transient physiological adaptations that facilitate the “fight-or-flight” response. This involves the concomitant activation of the sympatho-adrenal medullary (SAM) axis, resulting in the rapid release of catecholamines (adrenaline and noradrenaline) along with a transient rise in glucocorticoid levels. In contrast, chronic stress entails the sustained activation of the hypothalamic–pituitary–adrenal (HPA) axis, leading to prolonged cortisol secretion and downstream dysregulation of metabolic and neuroendocrine homeostasis (Karaer et al. 2023). Mobilization of brown adipose tissue (BAT) also plays a role in supporting acute stress and buffering the noxious effects of chronic stress by interacting with HPA. Cortisol binds to two types of specific intracellular receptors, inducing metabolic and behavioral changes that help the organism to cope with the stressor and restore allostatic balance (Romero and Wingfield 2015). However, prolonged or excessive cortisol production may have detrimental effects on the immune system, growth rate, and fertility (Hing et al. 2016). The quantification of cortisol levels provides valuable insights into the level of welfare of both humans and animals, including domestic and wild species.

Plasma is the most commonly used matrix for cortisol determination. Plasma cortisol levels rise rapidly after a stress event (within minutes) and decline approximately 20 min after the stressor ceases (Romero and Wingfield 2015). However, timing and magnitude of cortisol increases, or decline may differ in mammals. Plasma cortisol primarily reflects short-term stress responses and is strongly influenced by diurnal fluctuations (Dallman et al. 1993). Furthermore, in wild animals, plasma cortisol predominantly reflects the stress induced by capture (Romero and Wingfield 2015).

Alternative matrices, such as urine, feces, and hair, accumulate cortisol metabolite accumulation over longer time periods and are generally less affected by diurnal variation (Barukčić et al. 2025). These matrices are therefore more suitable for reflecting chronic and prolonged stress and for monitoring the influence of environmental variables on wild populations (Kalliokoski et al. 2019). Hair is a promising matrix due to its slow accumulation of cortisol, minimally-invasive sampling procedure, and stability at room temperature for months (Gow et al. 2010). Cortisol accumulates in the hair shaft over weeks to months via vascularization of the bulb and incorporation from sebaceous secretion and saliva during grooming (Cone 1996). In humans, it is estimated that 1 cm of hair length corresponds to approximately one month of cortisol accumulation (Russell et al. 2012) however, data are currently lacking for wild animal species (Barukčić et al. 2025).

Few studies have assessed hair cortisol in red and roe deer, and those that do often employ complex and expensive techniques such as radioimmunoassay (RIA) (Montillo et al., 2019; Ventrella et al. 2018) or lack specific validation (Dziki-Michalska et al. 2024a, 2024b). ELISA assays are more practical and cost-effective, as they do not require specialized facilities or waste management procedures. The demonstrated reliability of their analytical performance may support the use of ELISA as a practical and accessible alternative to RIA. High sensitivity expanded range ELISA test developed for salivary cortisol may be suitable for detecting low concentrations expected in hair, as already reported in humans (Meyer et al. 2014).

The European roe deer (*Capreolus capreolus*) is one of the most widespread cervid species in Italy and Europe. Although it can tolerate certain human-modified landscapes, such as agricultural areas, roe deer typically inhabit ecotones, transitional zones between meadows and forests (Perco 2011). Like other cervids, roe deer are highly sensitive to environmental stressors, including habitat fragmentation, human disturbance, and resource limitation. (Dziki-Michalska et al. 2023). Roe deer have increasing economic, cultural, and ecological importance, serving as a resource for game meat production and sport hunting, as well as representing a key component of European biodiversity (Burbaiteė and Csányi 2009). Moreover, as a solitary and territorial species, roe deer provide an excellent model for studying the effects of environmental variables on wildlife welfare.

In roe deer hair, cortisol levels and accumulation may depend on intrinsic factors (age and gender) or on extrinsic environmental stressors (type of environment, season). In particular, mating season (Dziki-Michalska et al. 2023) and competition with red deer have been associated to higher stress in male roe deer (Franchini et al. 2023).

Objectives.

The aims of this research are to propose an ELISA test for quantifying immunoreactive hair cortisol as an adequate method for monitoring long-term cortisol accumulation in roe deer. In particular we aim: (1) to evaluate the reliability of an ELISA kit, originally used for quantifying cortisol in saliva, to measure cortisol concentrations in roe deer hair; and (2) to investigate the effects of age and gender, (as these factors may influence roe deer hair cortisol levels), the type of environment, and seasonal variations (as they may act as stressors, thus functioning as a surrogate biological validation) on roe deer hair cortisol concentrations.

## Materials and methods

### Study site and animals

The roe deer included in this study were admitted to the Piacenza Wildlife Rescue Center for various reasons (Supplementary Table 1, Supplementary Fig. 1). The Center is located within an agricultural landscape less than 15 km from the city of Piacenza, at the foothills of the Apennine Mountains, and its operational area encompasses a range of environmental and ecological settings.Most samples were collected in 2021 (49%) and 2023 (27%), with one sample from 2020 and the remaining from 2022 (22%). A total of 45 European roe deer were analyzed, including 20 males and 25 females. Eight individuals were juveniles (< 1 year old), 11 were subadults (1–2 years old), and 13 were adults, while the age of the remaining 13 individuals was not available.Eleven samples (24%) were collected during the rutting season (July and August), 33 samples (73%) during the non-rutting months, and in one case the season was not recorded.

Twenty-one animals (46%) were rescued from agricultural environments, 14 (31%) from forested areas, and 2 (4%) from urban or peri-urban settings. In 9 cases (20%), the rescue location was either not adequately recorded or the type of environment could not be determined.

### Samplings

Each roe deer was submitted to clinical examination upon arrival at the Center. During clinical examination, hair samples from all the animals were collected by shaving a 2 × 2 cm area on the back (dorsal caudal region) with a razor and stored in plastic vials at −20 °C until extraction. All hair samples are derived from different animals. Metadata collected for each case included sex, age (adult, subadult, juvenile), type of environment (forest, agricultural, urban/peri-urban), date of sampling, and cause of rescue.

### Cortisol measurement

Cortisol extraction was performed according to Meyer et al. (2014) with some modifications. Briefly, the hair samples were washed twice with 7.5 mL of isopropanol to remove cortisol not incorporated into the hair shaft. After air-drying for 48 h, the samples were ground into a fine powder using a ball mill. Cortisol was extracted from 50 mg of pulverized hair in 1 mL of methanol, and the mixture was left to rotate overnight. After centrifugation, 0.7 mL of the supernatant was removed and dried using a vacuum rotary evaporator. The extract was then reconstituted in 200 µL of assay diluent and stored at −20 °C until analyses.

The ELISA test was performed using the Salimetrics^®^ Cortisol Enzyme Immunoassay Kit (Salimetrics LLC, PA, USA). All samples were run in duplicate. A standard curve was generated using standard solutions (3.0 to 0.12 nmol/L), and the curve was plotted to determine the best-fitting equation to calculate the concentration from optical densities. The concentrations were then converted to reflect the amount of hair powder and expressed as pg/mg. Two samples provided in the kit were used as controls. Analytical performances, reported by the manufacturer, are the following: precision: intra-assay CV = 4 to 7%; inter-assay CV = 3–11%; analytical sensitivity = 0.007 ug/dL; linearity = mean recovery 101% ± 4%, antibody specificity= less than 1% cross-reactivity for all other steroids except for dexamethasone (19.2%). The precision of the assay for roe deer hair cortisol was evaluated on six pool samples: for intra-assay CV, six pool samples were run in triplicate in the same plate. For inter-assay CV, six pool samples were run in two different sessions and plates. Both inter- and intra-assay coefficients of variation (CV) were calculated. The accuracy of the assay was assessed through spike and recovery tests, by mixing a pool sample with control material at different concentrations (100%, 75%, 50% and 25%), as serial dilutions with buffer (100%, 75%, 50% and 25%), and parallelism, by mixing variable amounts of two pool samples with different concentrations (80 + 20%, 60 + 40% 50 + 50% and 20 + 80%) (Westgard 2008).

### Statistical analysis

Statistical analyses were performed using R Studio (R version 4.2.2). The distribution of data was assessed using the Shapiro-Wilk test. Statistical differences between groups were assessed using the Kruskal-Wallis test (age and environment) or the Wilcoxon test (sex and season). Post hoc Dunnett test was used as a post-hoc test. Statistical significance was considered for *p* < 0.05. Parallelism was evaluated using a regression test.

### Ethical declaration

The roe deer included in this study were not captured for the specific aims of the research and hair sampling was minimally invasive, thus no specific ethical assessment protocol number was necessary according to the authors’ institution.

## Results

The reproducibility and repeatability of the method were reflected by an intra-assay coefficient of variation (CV) of 4.8% and an inter-assay CV of 14.5% (supplementary Table 2). Linearity, assessed through R-squared values, was 0.983 for the dilution test, 0.988 for the spike-and-recovery test, and 0.964 for parallelism by mixing two samples (Supplementary Figs. 2, 3, and 4).

**Fig. 1:**
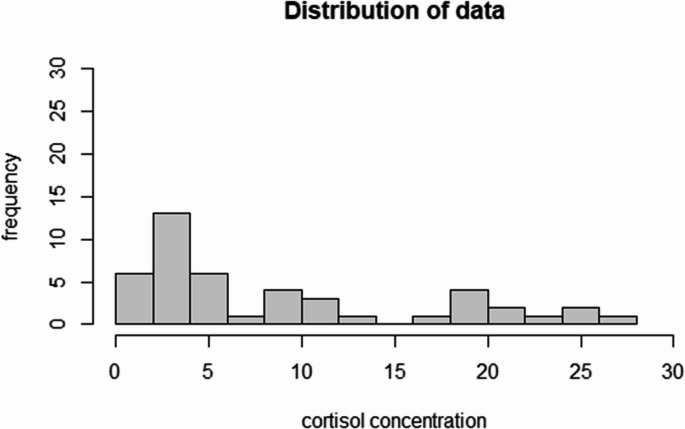
distribution of results of hair cortisol concentrations in roe deer. Results are expressed in pg/mg

Hair cortisol concentrations ranged from 0.7 pg/mg to 27.25 pg/mg with a non-normal, bimodal distribution (*p* = 0.001, Fig. 1). Overall, the median concentration of cortisol was 4.43 pg/mg (interquartile range-IQR = 2.81–14.55). Males showed numerically higher values, although not significant (*p* = 0.14), with median hair cortisol levels of 6.79 pg/mg (IQR: 3.57–19.65) vs. 3.8 pg/mg (IQR = 2.39–10.43 pg/mg) in females.

Adults (*n* = 13), regardless of sex, had a median cortisol concentration of 3.64 (IQR = 3.10–5.85).

Subadults (*n* = 11) had a median concentration of 9.95 pg/mg (IQR: 4.97–19.39.97.39).

Juveniles (*n* = 8) exhibited the highest median cortisol concentration at 18.50 pg/mg (IQR: 8.74–23.50).

**Fig. 2:**
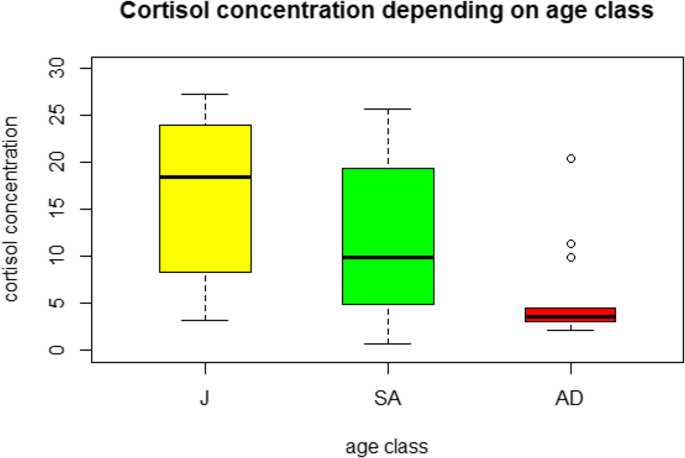
Cortisol concentrations in juveniles (J, *n* = 8), subadults (SA, *n* = 11), and adults (AD, *n* = 13). Values are expressed in pg/mg

Statistically significant differences in cortisol concentrations were observed among the age groups (*p* = 0.0029) (Fig. 2). Post hoc test confirmed the statistical difference between adults and juveniles (*p* = 0.025).

Regarding the effects of season subadults and juveniles did not show any statistical difference in animals sampled during the rutting season compared with those sampled outside the rutting season (*p* > 0.05).

**Fig. 3:**
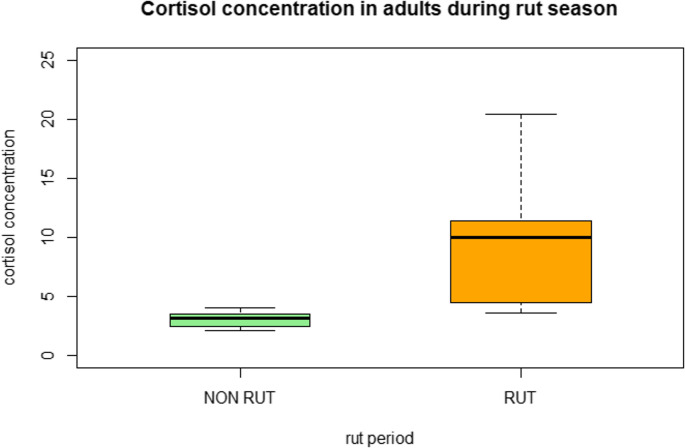
Cortisol concentrations in adult roe deer during the rutting season (RUT, *n* = 5) and non-rutting season (NON-RUT, *n* = 8). Values are expressed in pg/mg

However, when considering only adult individuals, those sampled during the rutting season had a median cortisol concentration of 9.98 pg/mg (IQR: 4.49–11.36), compared to 3.23 pg/mg (IQR: 2.44–3.53) in those sampled during the non-rutting season. This difference was statistically significant (*p* < 0.01) (Fig. 3).

All the other variables tested showed no statistically significant association with hair cortisol concentration. Specifically, Kruskall-Wallis test show no differences among the years of sampling (*p* = 0.16), between sexes (*p* = 0.14), and among the types of environment (*p* = 0.43).

## Discussion

The hair cortisol concentrations detected in roe deer are consistent with those reported using alternative analytical techniques (Ventrella et al. 2018). A recent study (Dziki-Michalska et al. 2024a, 2024b), however, reported lower concentrations using a different ELISA kit and an extraction protocol that did not include hair pulverization. As no validation data were available for the ELISA kit used in that study, it cannot be ruled out that both the different assays and the extraction procedures may have influenced cortisol recovery. Numerous extraction protocols and ELISA assays for salivary cortisol are currently available, with both potentially affecting the results. An effort to standardize hair cortisol measurement procedures in humans was proposed in 2015 (Russell et al. 2015), and although results across four different laboratories showed strong correlations, values were not directly interchangeable. While no such inter-laboratory studies have yet been conducted in animals and in cervids in particular, it is likely that similar outcomes would be observed across non-human species. It is recommended that results obtained using different assays or extraction methods should not be compared and that only general trend or patterns are compared as already suggested in previous studies (Palme 2019).

The distribution of overall results showed a non-normal, bimodal pattern, likely reflecting age-related differences. Juvenile roe deer exhibited significantly higher hair cortisol concentrations compared to adults. The cause of this difference is currently unknown, but it likely reflects higher levels of chronic stress due to weaning, nutritional challenges during the first winter, and low social status. Newborn roe deer are regularly left alone after birth, lying still among the vegetation, trusting just in their camouflage for their defense against predators. They are breastfed for approximately 2–3 months and remain with the mother for up to 10 months, or even longer (Perco 2011). To the authors’ knowledge, such age-related differences have not been previously reported in roe deer, but higher hair cortisol concentration in juveniles is expected considering the above-mentioned behavior. In addition, high hair cortisol concentration in juveniles has been reported in cows (González-de-la-Vara et al. 2011), and in horses (Comin et al. 2012). In contrast, Dziky-Michalska et al. (2024b) did not find any age-related difference in hunted male roe deer, but enrolment comprised animals with at least 2 years old. In addition, we cannot exclude that this difference is unrelated to stress condition, considering the myriad of effects of cortisol in metabolic events (MacDougall-Shackleton et al. 2019). Additionally, elevated cortisol in juveniles may reflect metabolic demands, growth, and activity, not only stress. Our findings suggest that age should be considered as a confounding factor when comparing groups with varying demographic compositions, including juveniles and subadults. More studies on the possible causes of stress in juvenile roe deer are needed.

No statistically significant differences were observed between males and females, although males exhibited slightly higher cortisol levels. These findings are consistent with previous researches in roe deer (Franchini et al. 2023) and other cervids (Caslini et al. 2016; Huber et al. 2003), and contrast with findings in other mammals where sex-based differences have been documented. This variability may be influenced by behavioral differences, body condition, and the metabolic effects of gonadal steroids (Heimburge et al. 2019). In the present study, no seasonal differences were observed when considering the full cohort of juveniles, subadults, and adults. However, when only adults were analyzed, higher cortisol concentrations were observed in samples collected during the rutting season. This finding is not unexpected, as adult roe deer exhibit strong territorial behavior, and the rutting season is likely to constitute a significant physiological and social stressor. Seasonal variations, such as the rutting period, may modulate cortisol concentrations in adults, whereas juveniles often show high baseline levels regardless of season, potentially masking seasonal effects. In adults, hormonal responses may also be influenced by territorial status, with dominant males exhibiting peak cortisol levels during the rut, while juveniles, being non-territorial, maintain elevated baseline levels. However, we cannot exclude that hair cortisol elevations may reflect physiological or metabolic demands rather than stress in itself. Several variables (physiological, pathological, and environmental) may influence cortisol-free concentration in plasma. By these, sexual steroids may alter cortisol plasma or cortisol binding globulin (CBG) concentrations (Bae and Kratzsch 2015), thereby altering cortisol hair accumulation. These variations have been also postulated in roe deer (Ventrella et al. 2018). Unfortunately, the small number of individuals sampled during this period, and the presence of only one adult female, limited the possibility of a sex-based comparison. Results from the literature are contradictory on this issue in roe deer, with a previous study (Franchini et al. 2023) failing to detect any significant difference in hair cortisol concentrations across months, and another recent study reporting higher plasma cortisol concentrations at the end of the mating and hunting seasons (Dziki-Michalska et al. 2024b). In other cervids, studies (Tallo-Parra et al. 2017; Prandi et al. 2018) reported elevated cortisol levels during late autumn and winter. However, roe deer biology is distinct because antler growth occurs in winter and mating takes place in summer, both of which may influence cortisol levels.

Conversely, no significant differences were found in hair cortisol concentrations when comparing different causes of rescue, types of environments, or years of sampling. This is expected since hair cortisol is not influenced by acute stressors such as vehicle collisions, predation, capture, or short-term illness (Heimburge et al. 2019). Although data on the kinetics of cortisol incorporation into hair in roe deer are lacking, it is plausible that factors such as species, body region sampled, and season (influencing hair growth and molting) may affect hormone deposition. Data on kinetics of cortisol accumulation in hair are not available for wild species, but an experimental study in goats demonstrated increased hair cortisol concentrations within one month following repeated ACTH administration (Endo et al. 2018). However, conducting a comparable study in wild roe deer would be ethically challenging. Ongoing research investigating animals rescued for medical reasons may help elucidate the period of stress represented by hair cortisol concentrations in this species.No statistically significant differences were observed among roe deer sampled from forested, agricultural, or peri-urban environments. However, the limited number of individuals from peri-urban areas may have influenced this result. A recent study by Franchini et al. (2023) investigating the effects of habitat suitability on hair cortisol levels in roe deer also found no significant association, suggesting that roe deer are able to adapt even to suboptimal habitats, provided they have access to nearby areas with adequate food and shelter. Future studies incorporating a larger sample size and spatial analyses using buffer zones corresponding to home ranges may help confirm these observations.

To the best of our knowledge, only 14 papers evaluated cortisol in roe deer, eight of which focused on fecal corticosteroid metabolites and four on plasma cortisol concentration. Of the two studies that assessed hair cortisol, one used a Radio-Immunoassay (RIA) (Ventrella et al. 2018), while the other (Dziky-Michalska et al. 2024a, 2024b) used a different ELISA, without reporting analytical validation results.

Hair cortisol concentrations were not significantly affected by the cause of rescue, environmental type, or year of sampling, reinforcing the notion that hair integrates chronic rather than acute stressors. This contrasts with plasma cortisol, which rises rapidly in response to immediate stress but is highly sensitive to capture and handling (Sheriff et al. 2011). Fecal corticosteroid metabolites provide intermediate temporal resolution but are subject to environmental and dietary influences. Hair offers the advantage of stability at room temperature, minimal invasiveness, and integration of cortisol over weeks to months, making it a suitable matrix for assessing chronic stress in wild populations.

The ELISA kit used in the present project proved to be a reliable method for measuring hair cortisol in roe deer, representing a cost-effective and practical alternative to more complex techniques such as LC–MS/MS or RIA. The assay showed acceptable accuracy and precision; however, the moderately high inter-assay variability indicates the need to analyze samples in batches and to include appropriate control samples across different runs. The extraction procedures allowed cortisol quantification within the linear range of the ELISA kit starting from as little as 50 mg of hair, although concentrations were generally close to the lower detection limit. Critical steps in the protocol include hair grinding using a ball mill, which may lead to sample loss, and the precise weighing of pulverized hair, requiring an analytical balance with high resolution and repeatability. While these methodological considerations confirm the feasibility of the approach, several limitations of the study must be acknowledged. The sample size was limited, and in some cases, metadata were incomplete, preventing robust statistical comparisons across subgroups. This result may have been influenced by the limited number. Despite these limitations, obtaining standardized samples from wild animals is challenging, and most studies on this topic have reported similar caseloads. Furthermore, the lack of data on the kinetics of cortisol incorporation into hair impedes the ability to draw definitive conclusions regarding the temporal relationship between stress events and hormone accumulation. An effective demonstration of the timing of accumulation of cortisol in hair shaft should include a physiological validation through serial ACTH injections and hair cortisol monitoring. This procedure in a wild species is complex and ethically unacceptable. However, rut season can be considered a major stressor for this territorial species. By comparing hair cortisol levels of adult animals during rut with those in other seasons, we observed an increase, supporting its use as a marker of chronic stress. Interestingly, the increase in hair cortisol was not found in the months of September and October (before molting). This supports the hypothesis that hair cortisol reflects more a short to intermediate stressor lasting for weeks to a few months than a progressive accumulation due to a chronic stress (Kalliokoski et al. 2019).

An extension of caseload would help confirm our results and potentially identify other sources of stress and ecologically relevant stressors in wild roe deer.

## Conclusions

Hair cortisol appears to be a promising biomarker for assessing chronic stress, even though measuring glucocorticoid concentrations alone is not sufficient to fully characterize the vertebrate stress response (Breuner et al. 2013) in wild roe deer. This method has potential applications in wildlife welfare monitoring, population management for conservation, disease prevention, and ensuring the quality of game meat. It could also facilitate the evaluation of environmental impacts, including interactions with conspecifics, other species (e.g., red deer, wild boar), predators (e.g., wolves), and anthropogenic pressures.

## Supplementary Information

Below is the link to the electronic supplementary material.

Supplementary Material 1

## Data Availability

Raw data are available upon reasonable request to the authors institution.
