# Supplementary material for: Hair cortisol concentrations as marker of chronic stress in wild roe deer (Capreolus capreolus)
Source: Vet Res Commun. 2026 Feb 7;50(2):148. doi: 10.1007/s11259-026-11106-6 (PMC12882847; doi:10.1007/s11259-026-11106-6)
Supplement: Supplementary file 1 — Supplementary Material 1 [file 11259_2026_11106_MOESM1_ESM.docx]

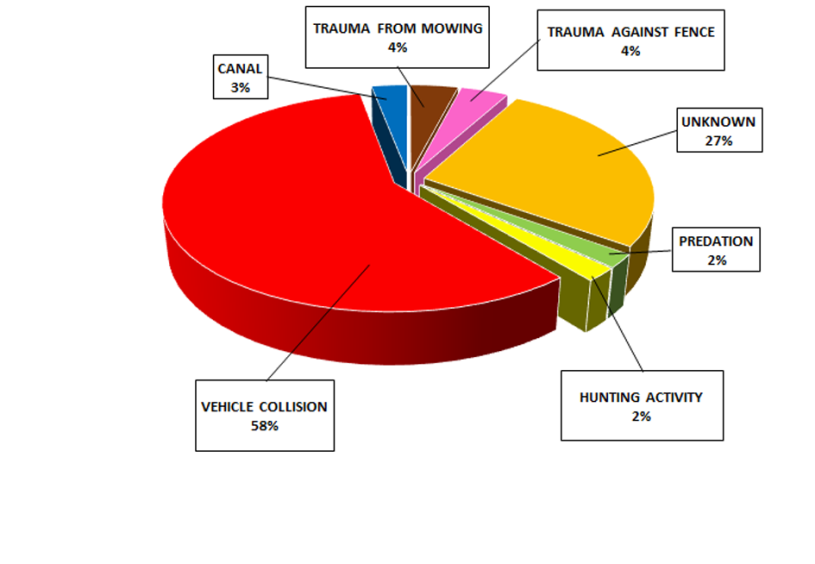


***Supplementary Figure 1****:* *Causes of recovery for the 45 roe deer included in this study, illustrated as a pie chart.*

| **ID** | **YEAR** | **SEX** | **AGE** | **MONTH** | **RUT SEASON** | **AREA** |
| --- | --- | --- | --- | --- | --- | --- |
| **1** | 2020 | F | UNK | DEC | NON RUT | FOREST |
| **2** | 2021 | F | UNK | JAN | NON RUT | UNK |
| **3** | 2021 | F | UNK | APR | NON RUT | UNK |
| **4** | 2021 | F | AD | JUN | NON RUT | AGRICULTURAL |
| **5** | 2021 | F | J | JUN | NON RUT | FOREST |
| **6** | 2021 | F | J | JUN | NON RUT | FOREST |
| **7** | 2021 | F | J | JUN | NON RUT | FOREST |
| **8** | 2021 | M | J | JUN | NON RUT | FOREST |
| **9** | 2021 | M | J | JUN | NON RUT | AGRICULTURAL |
| **10** | 2021 | F | SA | JUN | NON RUT | AGRICULTURAL |
| **11** | 2021 | M | SA | JUN | NON RUT | AGRICULTURAL |
| **12** | 2021 | F | AD | JUL | RUT | AGRICULTURAL |
| **13** | 2021 | M | AD | JUL | RUT | AGRICULTURAL |
| **14** | 2021 | F | SA | JUL | RUT | AGRICULTURAL |
| **15** | 2021 | M | AD | AUG | RUT | AGRICULTURAL |
| **16** | 2021 | M | J | AUG | RUT | AGRICULTURAL |
| **17** | 2021 | M | SA | AUG | RUT | AGRICULTURAL |
| **18** | 2021 | F | J | SEP | NON RUT | URBAN |
| **19** | 2021 | F | UNK | SEP | NON RUT | UNK |
| **20** | 2021 | F | AD | NOV | NON RUT | AGRICULTURAL |
| **21** | 2021 | F | SA | NOV | NON RUT | UNK |
| **22** | 2021 | F | UNK | NOV | NON RUT | FOREST |
| **23** | 2021 | F | UNK | NOV | NON RUT | FOREST |
| **24** | 2022 | M | AD | JUL | RUT | UNK |
| **25** | 2022 | F | UNK | JUL | RUT | AGRICULTURAL |
| **26** | 2022 | M | AD | AUG | RUT | UNK |
| **27** | 2022 | M | SA | AUG | RUT | FOREST |
| **28** | 2022 | M | UNK | AUG | RUT | AGRICULTURAL |
| **29** | 2022 | F | AD | SEP | NON RUT | UNK |
| **30** | 2022 | F | J | SEP | NON RUT | FOREST |
| **31** | 2022 | M | SA | SEP | NON RUT | AGRICULTURAL |
| **32** | 2022 | F | UNK | SEP | NON RUT | AGRICULTURAL |
| **33** | 2022 | F | AD | UNK | UNK | UNK |
| **34** | 2023 | M | SA | JAN | NON RUT | AGRICULTURAL |
| **35** | 2023 | F | AD | FEB | NON RUT | AGRICULTURAL |
| **36** | 2023 | M | AD | FEB | NON RUT | AGRICULTURAL |
| **37** | 2023 | F | UNK | FEB | NON RUT | FOREST |
| **38** | 2023 | F | AD | MAR | NON RUT | FOREST |
| **39** | 2023 | F | SA | MAR | NON RUT | AGRICULTURAL |
| **40** | 2023 | M | AD | APR | NON RUT | FOREST |
| **41** | 2023 | M | SA | APR | NON RUT | FOREST |
| **42** | 2023 | M | SA | APR | NON RUT | UNK |
| **43** | 2023 | M | UNK | APR | NON RUT | AGRICULTURAL |
| **44** | 2023 | M | UNK | APR | NON RUT | FOREST |
| **45** | 2023 | M | UNK | APR | NON RUT | URBAN |

***Supplementary table 1****:data from 45 roe deer included in the present study. AD = Adult; SA = Subadult; J = Juvenile; UNK = Unknown, RUT period considered as July–August based on regional breeding phenology.*

| **INTERASSAY CV** | | **Mean CV** | **4,8%** |  |  |  |
| --- | --- | --- | --- | --- | --- | --- |
| **pool #** | **well 1** | **well 2** | **well 3** | **Mean** | **St Dev** | **CV** |
| 1 | 3,18 | 3,34 | 3,60 | 3,37 | 0,21 | 6,3% |
| 2 | 6,56 | 6,53 | 6,15 | 6,41 | 0,23 | 3,6% |
| 3 | 6,01 | 6,26 | 6,60 | 6,29 | 0,29 | 4,7% |
| 4 | 7,09 | 7,09 | 6,80 | 6,99 | 0,17 | 2,4% |
| 5 | 2,04 | 2,05 | 2,30 | 2,13 | 0,15 | 7,0% |
| 6 | 7,90 | 7,30 | 7,92 | 7,71 | 0,35 | 4,6% |
|  |  |  |  |  |  |  |
| **INTRA ASSAY CV** | | **Mean CV** | **14,5%** |  |  |  |
| **pool #** | **plate 1** | **plate 2** | **Mean** | **St Dev** | **CV** |  |
| 1 | 5,04 | 6,38 | 5,71 | 0,95 | 16,6% |  |
| 2 | 1,90 | 1,04 | 1,47 | 0,61 | 41,5% |  |
| 3 | 9,71 | 8,63 | 9,17 | 0,76 | 8,3% |  |
| 4 | 9,90 | 8,64 | 9,27 | 0,89 | 9,6% |  |
| 5 | 12,66 | 11,91 | 12,29 | 0,53 | 4,3% |  |
| 6 | 5,55 | 6,07 | 5,81 | 0,37 | 6,3% |  |

***Supplementary table 2:*** *results of precision: for inter-assay CV, six pool samples were run in triplicate in the same plate. For intra-assay CV, six pool samples were run in two different sessions and plates.*

**
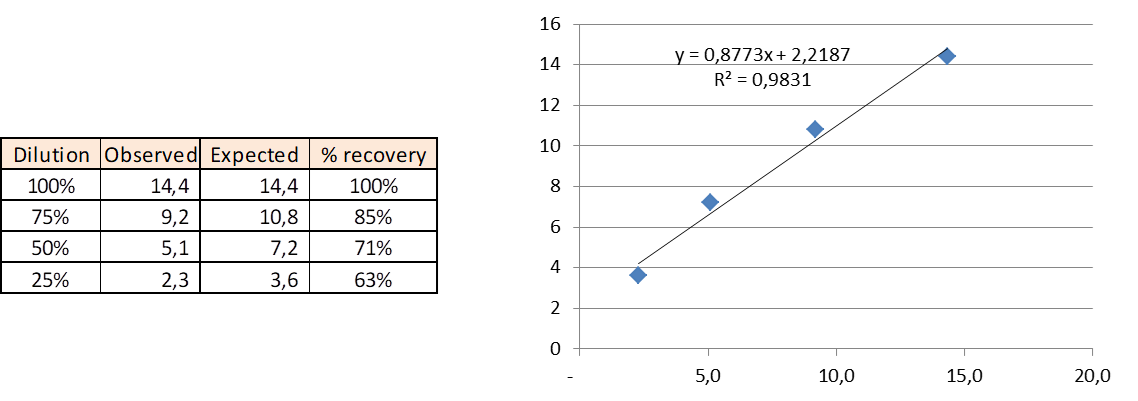
**

***Supplementary figure 2: results of serial dilution with buffer (P<0.001)***

**
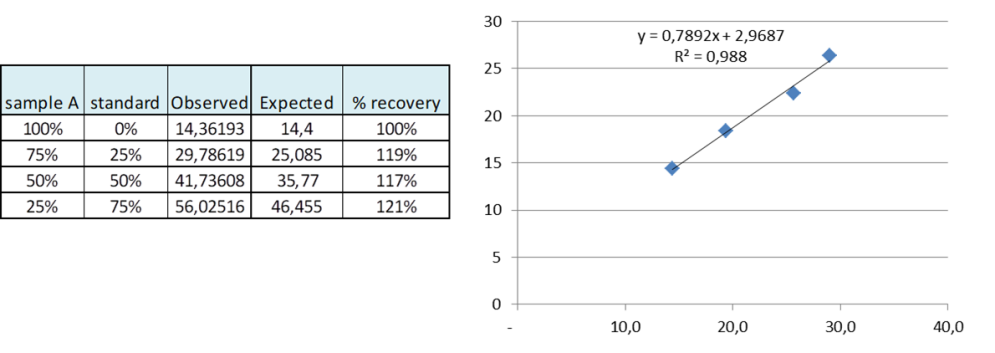
**

***Supplementary figure 3: results of spike and recovery obtained by adding cortisol standard solution to one sample (14.4 pg/mg) (p<0.001)***

***
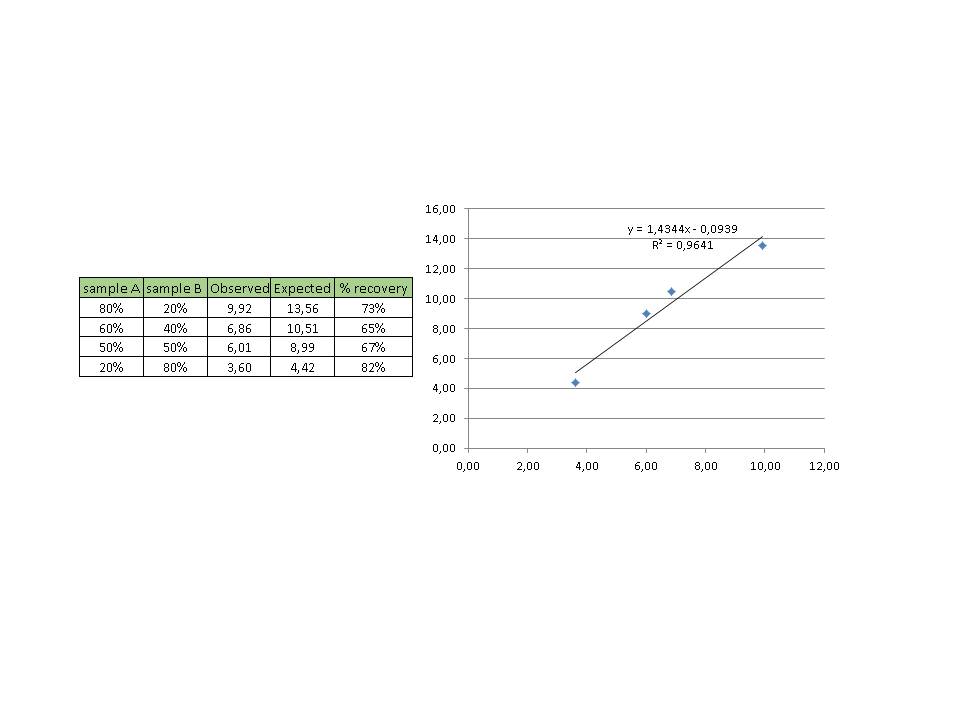
***

***Supplementary figure 4: Parallelism test by mixing different concentration of two samples (16.61 and 1.37 pg/mg respectively) (p<0,01)***
